# Diabetes Type 2 in Women: The Impact of EndogenousOestrogen, A Population‐Based Study With About 2 Decades of Follow‐Up

**DOI:** 10.1002/edm2.70267

**Published:** 2026-08-31

**Authors:** Fahimeh Ramezani Tehrani, Maryam Mousavi, Mahbanoo Farhadi‐Azar, Fatemeh Mahboobifard, Fereidoun Azizi, Maryam Farahmand

**Affiliations:** ^1^ Reproductive Endocrinology Research Center, Research Institute for Endocrine Molecular Biology, Research Institute for Endocrine Sciences Shahid Beheshti University of Medical Sciences Tehran Iran; ^2^ Foundation for Research & Education Excellence Vestavia Hills Al USA; ^3^ Department of Pharmacology, School of Medicine Shahid Beheshti University of Medical Sciences Tehran Iran; ^4^ Endocrine Research Center, Research Institute for Endocrine Disorders, Research Institute for Endocrine Sciences Shahid Beheshti University of Medical Sciences Tehran Iran

**Keywords:** endogenous oestrogen, menarche, menopause, the incidence of diabetes mellitus

## Abstract

**Aims:**

The role of endogenous oestrogen exposure (EEE) in type 2 diabetes (T2D) remains understudied despite its potential importance. This study aimed to assess T2D incidence in women with different endogenous oestrogen durations.

**Materials and Methods:**

At the initiation of the study, 6273 post‐menarche women above 20 years were included. After applying the exclusion criteria, 3,411 women remained (2,754 premenopausal and 657 menopausal). Each woman's EEE was determined, and they were monitored for the incidence of T2D. Based on the EEE duration, hazard ratios (HRs) and 95% confidence intervals (CI) for the T2D event were reported using Cox proportional hazards regression models.

**Results:**

The mean age and menarcheal age (standard deviation) of participants were 39.3(12.5) and 13.5(1.5) years, respectively. Finally, 30.9% (1053) of the women developed diabetes (819 (29.7%) premenopausal and 234 (35.6%) menopausal). The age and BMI‐adjusted model's findings indicated that, overall, the EEE z‐score was inversely associated with T2D incidence in postmenarcheal women [HR 0.91, 95% CI 0.85–0.97], meaning that the risk of T2D decreased by 9% for every 1‐SD increase in the EEE z‐score. Furthermore, after full adjustment for potential confounders, the association remained statistically significant (HR = 0.90, 95% CI: 0.84–0.96).

**Conclusions:**

The findings suggest that longer EEE duration may reduce the risk of T2D, emphasising the importance of reproductive history in health assessments.

## Graphical abstract

Shorter durations of endogenous oestrogen may increase the risk of type 2 diabetes. Reproductive history should be considered when managing type 2 diabetes.

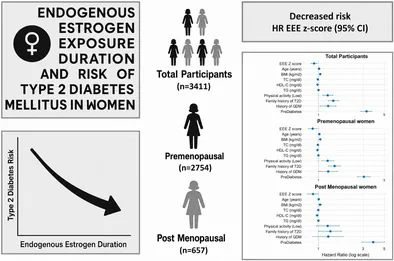

## 1 Introduction

Type 2 Diabetes (T2D) is a leading contributor to morbidity and mortality, placing substantial strain on public health resources worldwide in the 21st century [1]. It ranks as the eighth leading cause of death and disability globally [2]. According to the most recent national survey, 15.14% of Iranians aged 25 and older had diabetes in 2021. Without effective prevention, the number of cases is projected to reach 9.2 million [3, 4].

There is a gender difference in the incidence of diabetes before and after the reproductive period. This difference is influenced by hormonal changes during the reproductive years and exacerbated by socio‐economic and behavioural factors after menopause. Compared to reproductive‐age women, men tend to have a higher prevalence of diabetes due to biological predispositions such as greater visceral fat accumulation. After menopause, the risk for women significantly increases, surpassing that of men in older age groups [5]. Oestrogen provides a protective effect on glucose metabolism, thereby reducing the risk of diabetes in women during their reproductive years. Following menopause, the decline in oestrogen levels is linked to increased insulin resistance and a greater propensity for central adiposity, both of which contribute to a heightened risk of developing diabetes. Epidemiological studies have shown that women who experience early menopause (before the age of 40) face a significantly higher risk of developing type 2 diabetes compared to those who undergo menopause after the age of 50. This increased risk is primarily attributed to prolonged oestrogen deficiency, a phenomenon also noted in women who have undergone surgical oophorectomy [6, 7, 8]. The relationship between reproductive factors and diabetes remains unclear due to inconsistent findings. While breastfeeding seems to be protective and abortion may increase risk, results concerning age at menarche and menopause are conflicting [9, 10, 11, 12]. Furthermore, the cumulative effect of reproductive factors, as estimated by endogenous oestrogen exposure (EEE), on the incidence of T2D has not been thoroughly investigated, although its protective role against certain non‐communicable diseases related to T2D has been previously documented [13, 14, 15, 16].

To our knowledge, no prior study has examined the relationship between T2D incidence and EEE duration. In this research, we used data from the Tehran Lipid and Glucose Study (TLGS), a population‐based cohort with an average follow‐up of two decades, to examine this relationship.

The research used data from the Tehran Lipid and Glucose Study (TLGS), a population‐based cohort study.

## 2 Materials and Methods

### 2.1 Subjects

Our study utilised data from the TLGS, a population‐based cohort study established in 1998. Previous publications have described the TLGS [17, 18]. In brief, the study initially included 15,005 participants aged 3 years and older, comprising both males and females. A multistage cluster sampling method was employed to select participants. The primary objective of the TLGS was to examine the prevalence, incidence, risk factors, and characteristics of noncommunicable diseases (NCDs) within an urban population.

Data on demographic, anthropometric, reproductive, and metabolic traits, along with general medical examinations and laboratory assessments, were recorded, with follow‐ups every three years. Seven study phases have been completed, including the baseline and six subsequent follow‐ups. After nearly two decades, the collection of measurement variables has successfully concluded; however, the documentation of certain variables, such as cardiovascular events, continues. Participants were selected from an urban area.

**FIGURE 1:**
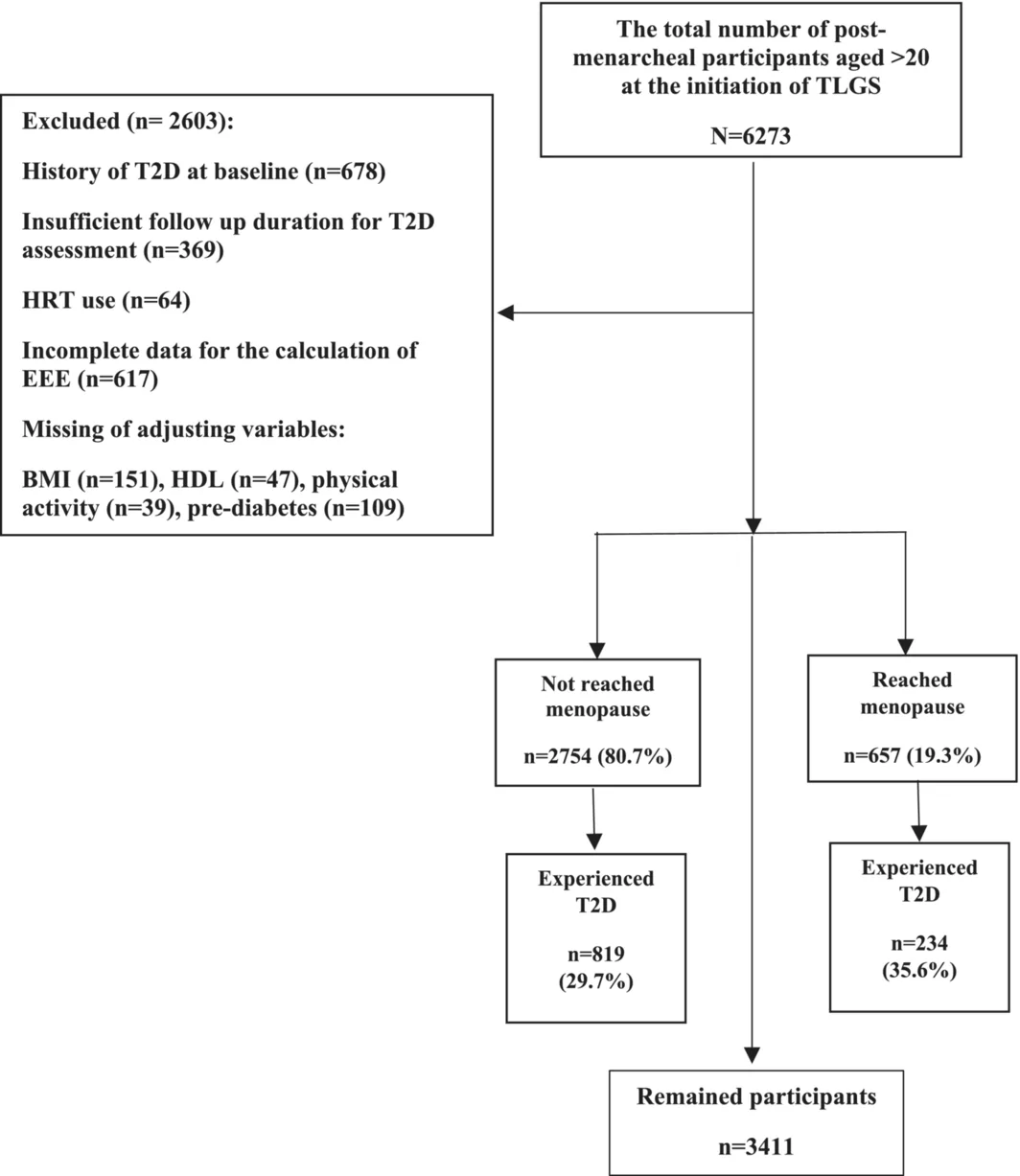
Study the flow chart. HDL‐c, High density lipoprotein; HRT, hormone replacement therapy; T2D, Type 2 diabetes; TLGS, Tehran lipid and glucose study.

For this study, we included all postmenarchal women aged 20 or older at baseline, regardless of time since menarche, who had at least one follow‐up visit (*N* = 6273). We excluded participants with a history of T2D at baseline, those with insufficient follow‐up (*n* = 369), those undergoing hormone replacement therapy (HRT) (*n* = 64), those lacking adequate information on EEE duration (*n* = 617), those missing data for adjustment variables (*n* = 347), and those who had undergone surgical menopause (*n* = 588). The study flowchart is presented in Figure 1. The ethics committee of the Research Institute for Endocrine Sciences approved the study (IR.SBMU.ENDOCRINE.REC. 1404.008). Written informed consent was obtained from all participants before the study commenced.

### 2.2 Measurements

Trained staff gathered extensive data on demographic details, lifestyle factors, and various risk factors associated with NCDs. This information was collected through face‐to‐face interviews and a standardised questionnaire. Additionally, all participants' heights and weights were measured using calibrated equipment and standardised procedures. These measurements were taken while participants stood, wearing minimal clothing and no shoes.

BMI is calculated by dividing weight in kilograms by the square of height in metres (kg/m^2^). Waist circumference is measured at the level of the umbilicus during mild expiration, positioned halfway between the lower rib edge and the iliac crest. Hip circumference is measured using an unstretched tape to the nearestcentimetre.

The Modifiable Activity Questionnaire was used to assess participants' physical activity levels. Participants who recorded fewer than 600 metabolic equivalent task minutes per week were classified as belonging to the low physical activity group [19].

Blood samples were collected following a 12‐ to 14‐h overnight fast between 7:00 and 9:00 AM. The blood samples were analysed on the same day. Further details about the laboratory measurements have been published elsewhere [17, 18].

### 2.3 Definition of Terms

#### 2.3.1 T2D Outcome

According to the American Diabetes Association (2014), T2D is defined as fasting blood glucose levels of ≥ 126 mg/dL (7.0 mmol/L), 2‐h plasma glucose (2hPG) ≥ 200 mg/dL, or the use of antidiabetic medication. Additionally, pre‐diabetes is defined as fasting plasma glucose between 100 mg/dL (5.6 mmol/L) and 125 mg/dL (6.9 mmol/L), or impaired fasting glucose, or a 2‐h plasma glucose level of 140 mg/dL (7.8 mmol/L) to 199 mg/dL (11.0 mmol/L) (impaired glucose tolerance) during the 75‐g oral glucose tolerance test or drug treatment [20].

#### 2.3.2 Exposure

The duration of EEE was defined as the interval between the age at menarche and the age at menopause, the age at incident T2D, or the end of follow‐up, whichever occurred first. To exclusively assess the estradiol‐dominant (follicular) phases of menstrual cycles, we subtracted the cumulative duration of pregnancies (40 weeks per birth or 20 weeks per abortion), hormonal contraceptive use (such as oral contraceptive), breastfeeding (number of months per child), and the progesterone‐dominant (luteal) phases of menstrual cycles (2 weeks per menstrual cycle) from the primary EEE variable [13, 14, 15, 16, 21]. The World Health Organization defines natural menopause as the complete cessation of menstrual flow for over 12 consecutive months, with no other underlying pathological or physiological causes [22]. Traditionally, it has been accepted that menopause occurs one year before the onset of 12 months free of periods.

### 2.4 Statistical Analysis

The normality of all continuous variables was assessed using a one‐sample Kolmogorov–Smirnov test. Data are presented as mean (SD) and median (interquartile range 25%–75%) for variables with normal and skewed distributions, respectively. Based on normality test results, *t*‐tests and Mann–Whitney tests are used to compare continuous variables between final event groups. Numbers and percentages represent categorical variables and are compared between final event groups using the Chi‐square test or Fisher's exact test (when at least one cell has sparse data).

The Cox proportional hazards regression model was used to assess the relationship between the EEE Z score and the risk of T2D, reporting hazard ratios (HRs) and 95% confidence intervals (CIs). The event date was defined as the day the incident T2D occurred, and the age at T2D onset was calculated. Women were censored or considered lost to follow‐up if they had not experienced the final event by the end of the follow‐up period. The interval between age at entry and age at T2D onset (in years) was used to calculate survival time for women with T2D incidence. The survival time was also determined for censored participants as the interval between menarche and the last follow‐up.

Cox regression models served as our primary statistical method to assess the effect of EEE Z score on T2D incidence. After conducting our analyses on the total sample (*n* = 3411), we performed subgroup analyses on natural menopausal women (*n* = 657) and premenopausal women (*n* = 2754). All models are reported as adjusted for age and BMI, and the model is fully adjusted. The multivariate Cox model incorporated age, BMI, TC, HDL‐C, TG, physical activity, history of pre‐DM, family history of T2D, and history of GDM as potential confounding variables. All adjustment variables are utilised at the study's baseline.

STATA version 14 SE (StataCorp, TX, USA) was used for all analyses, and a 2‐tailed *p*‐value of less than 0.05 was considered significant.

## 3 Results

Table 1 presents baseline characteristics of the study participants and compares them by T2D status.

**TABLE 1:** Baseline characteristics of the study participants' status stratified by T2D status at follow‐up.

Variables	Total (*N* = 3411)	Diabetes at follow‐up		p
		No (2,358)	Yes (*n* = 1053)	
Age, years^a^	39.3 (12.5)	38.3 (12.8)	41.8 (11.6)	< 0.001
Age at menarche, years^a^	13.5 (1.5)	13.5 (1.4)	13.4 (1.6)	0.08
Menopausal status^c^				
Reproductive age	2,754 (80.7)	1,935 (82.1)	819 (77.8)	0.003
Menopaused	657 (19.3)	423 (17.9)	234 (22.2)	
Age at menopause^a^, ^d^	50.01 (5.4)	50.1 (5.3)	49.7 (5.6)	0.39
BMI, kg/m^2^ ^a^	27.4 (4.8)	26.8 (4.6)	28.8 (4.9)	< 0.001
Marital status^c^				
Never married	131 (5.7)	131 (5.6)	37 (3.5)	0.011
Ever married	2,227 (94.4)	2,227 (94.4)	1,016 (96.5)	
Smoking status,^c^				
Never smoker	3,260 (95.6)	2241 (95)	1,019 (96.8)	0.23
Ever smokers	151 (4.4)	117 (5)	34 (3.2)	
Educational level,^c^				
< 12 years	3,054 (89.5)	2,088 (88.5)	966 (91.7)	0.005
> = 12 years	357 (10.5)	270 (11.5)	87 (8.3)	
*p*hysical activity^c^				
Low	2,275 (66.7)	1,551 (65.8)	724 (68.8)	0.088
High	1,136 (33.3)	807 (34.2)	329 (31.2)	
HDL‐C, mg/dl^a^	44.7 (11.2)	45.4 (11.4)	43.2 (10.4)	< 0.001
TC, mg/dl^a^, ^c^	205.6 (46.4)	201.5 (44.97)	214.6 (48.5)	< 0.001
TG, mg/dl^b^	126 (87, 182)	116 (82, 169)	151 (105, 210)	< 0.001
Comorbidities				
Pre Diabetes^c^	691 (20.0)	275 (11.7)	416 (39.5)	< 0.001
History of GDM^c^	294 (8.6)	169 (7.2)	125 (11.9)	< 0.001
Family history of T2D^c^	909 (26.6)	543 (23)	366 (34.8)	< 0.001
Total EEE duration, weeks^b^	720.7 (603.2, 823.9)	735.1 (639.5837.1)	717.7 (585.9816.3)	0.02

Abbreviations: BMI, body mass index; CVD, cardiovascular disease; EEE, endogenous oestrogen exposure; GDM, gestational diabetes mellitus; SD, standard deviation; T2D, type 2 diabetes; TC, total cholesterol; TG, triglyceride.

^a^ Values are presented as mean (SD).

^b^ Median (IQR 25%–75%).

^c^ Percentage As appropriate.

^d^ Just for menopausal participants.

Participants' mean (SD) ages were 39.3 (12.5) years, 34.86 (8.91) years, and 58.21 (6.50) years in the postmenopausal group. Age at menarche and baseline BMI were 13.5 (1.5) years and 27.4 (4.8) kg/m^2^, respectively. In a comparison of participants' characteristics, women with T2D had a significantly higher mean age (*p*‐value < 0.001), BMI (*p* value < 0.001), total cholesterol (TC) (*p* value < 0.001), and triglycerides (TG) (*p*‐value < 0.001) than those without T2D. Furthermore, the mean HDL‐C was lower in women with T2D (*p*‐value < 0.001). At baseline, several characteristics differed significantly between women who developed T2D during follow‐up and those who did not (Table 1). Women who developed T2D were older, had higher BMI, and showed a less favourable lipid profile, including higher total cholesterol and triglycerides and lower HDL‐C. They also had a higher prevalence of prediabetes, a history of gestational diabetes, and a family history of T2D. Differences were also observed in menopausal status and educational level.

Table 2 displays the HRs for the effect of the EEE z‐score on T2D incidence among study participants. The results from the adjusted model, which accounted for age and BMI, indicated that the EEE z‐score was inversely associated with the incidence of T2D (adjusted HR 0.91, 95% CI 0.85–0.97; *p* = 0.003), suggesting that for every 1‐SD increase in the EEE z‐score, the hazard of T2D declined by 9%. These findings remained consistent after adjusting for age, BMI, TC, HDL‐C, TG, physical activity, history of pre‐DM, family history of T2D, and history of GDM (HR 0.90, 95% CI 0.84–0.96; *p* = 0.002).

**TABLE 2:** Hazard ratios and 95% confidence intervals (CI) from the unadjusted and adjusted analysis of the association between EEE with T2D incidence stratified by menopause status.

Model	Variables	Total participants survival time median (Q25‐Q75) 17.64 (12.86‐19.58) (*N* = 3411)		Premenopausal women Survival time median (Q25‐Q75) 18.44 (12.99‐19.74) (*n* = 2754)		Menopausal women survival time median (Q25‐ Q75) 12.74 (7.23 _18.64) (*n* = 657)	
		HR (95% CI)	*p*	HR (95% CI)	*p*	HR (95% CI)	*p*
Model 1	EEE Z score	0.91 (0.85, 0.97)	0.003	0.84 (0.76, 0.93)	0.001	0.93 (0.82, 1.06)	0.310
				Adjusted model			
Model 2	EEE Z score	0.90 (0.84, 0.96)	0.002	0.87 (0.79, 0.96)	0.006	0.88 (0.77, 1.00)	0.06
	Age, years	1.02 (1.01, 1.03)	< 0.001	1.03 (1.02, 1.04)	< 0.001	1.01 (0.99, 1.03)	0.21
	BMI, kg/m^2^	1.05 (1.04, 1.06)	< 0.001	1.05 (1.03, 1.07)	< 0.001	1.04 (1.01, 1.08)	0.003
	TC, mg/dl	0.99 (0.98, 1.00)	0.323	0.999 (0.997, 1001)	0.254	1.00 (0.99, 1.01)	0.757
	HDL‐C, mg/dl	0.98 (0.97, 0.99)	< 0.001	0.989 (0.982, 0.996)	0.004	0.97 (0.96, 0.99)	< 0.001
	TG, mg/dl	1.001 (1.00, 1.001)	0.07	1.001 (1.00, 1.002)	0.02	1.00 (0.99, 1.001)	0.587
	Physical activity (Low)	1.26 (1.10, 1.43)	0.001	1.26 (1.08, 1.46)	0.003	1.26 (0.95, 1.67)	0.115
	Family history of T2D (Ref: NO)	1.44 (1.27, 1.64)	< 0.001	1.49 (1.29, 1.72)	< 0.001	1.34 (1.01, 1.76)	0.039
	History of GDM (Ref: NO)	1.27 (1.05, 1.54)	0.016	1.27 (1.01, 1.6)	0.044	1.27 (0.88, 1.83)	0.205
	Pre Diabetes (Ref: NO)	3.26 (2.85, 3.73)	< 0.001	3.06 (2.61, 3.58)	< 0.001	3.85 (2.92, 5.06)	< 0.001

*Note:* Model 1: Age and BMI adjusted. Model 2: Fully adjusted. *p*‐value < 0.05 is statistically significant.

Abbreviations: BMI, body mass index; CI, confidence interval; EEE, endogenous oestrogen exposure; GDM, gestational diabetes mellitus; HDL‐C, high‐density lipoprotein cholesterol; HR, hazard ratio; Ref, reference; T2D, type 2 diabetes; TC, total cholesterol; TG, triglycerides.

The findings of subgroup analyses demonstrated that the EEE z‐score was inversely associated with the incidence of T2D in the subgroup of premenopausal women (age‐ and BMI‐adjusted HR 0.84, 95% CI 0.76–0.93; *p* = 0.001), indicating that for every 1‐SD increase in the EEE z‐score, the risk of T2D declined by 16%. The results remained consistent following adjustments for age, BMI, TC, HDL‐C, TG, physical activity, history of pre‐DM, family history of T2D, and history of GDM (HR 0.87, 95% CI 0.79–0.96; *p* = 0.006).

In the subgroup of menopausal women (*N* = 657), the association approached but did not reach statistical significance (adjusted HR = 0.88, 95% CI: 0.77–1.00; *p* = 0.06). The confidence interval included 1.0, indicating that the association approached but did not reach conventional statistical significance.

**FIGURE 2:**
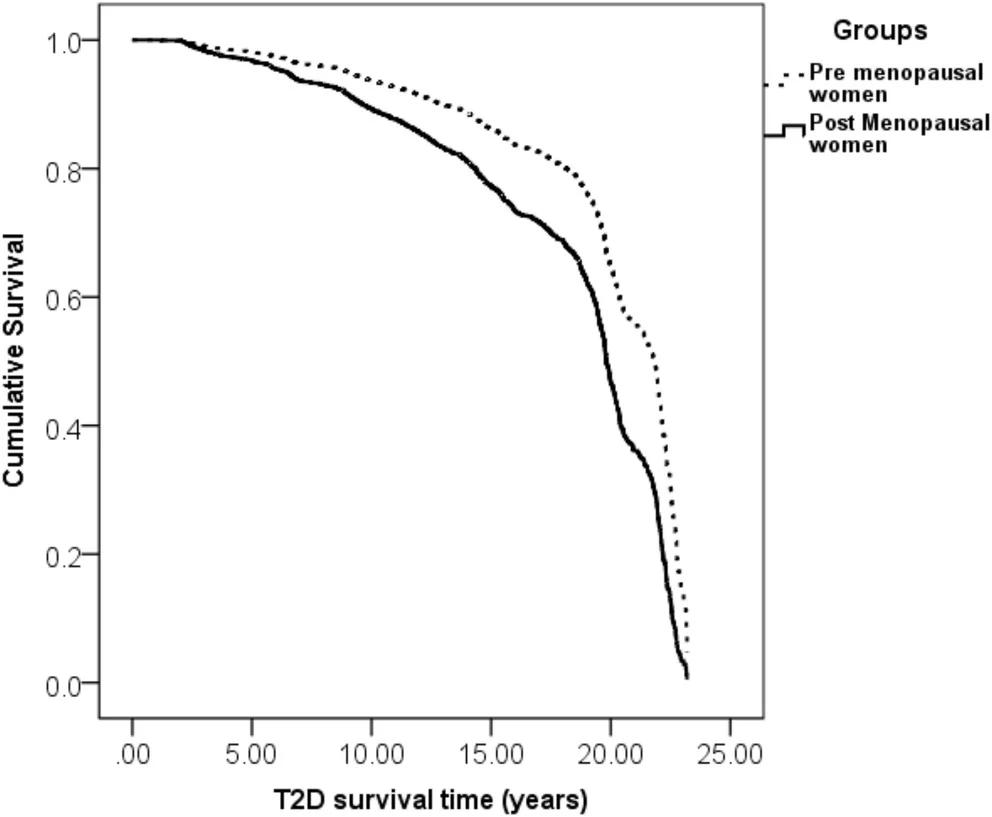
Adjusted Cox model–Based survival curves for type 2 diabetes incidence across EEE levels in Pre and Postmenopausal Women.

Figure 2 presents adjusted survival curves derived from the Cox proportional hazards model, showing cumulative T2D‐free survival by menopausal status. After adjustment for potential confounders, postmenopausal women exhibited consistently lower cumulative survival probabilities than premenopausal women, with a widening separation over time.

**FIGURE 3:**
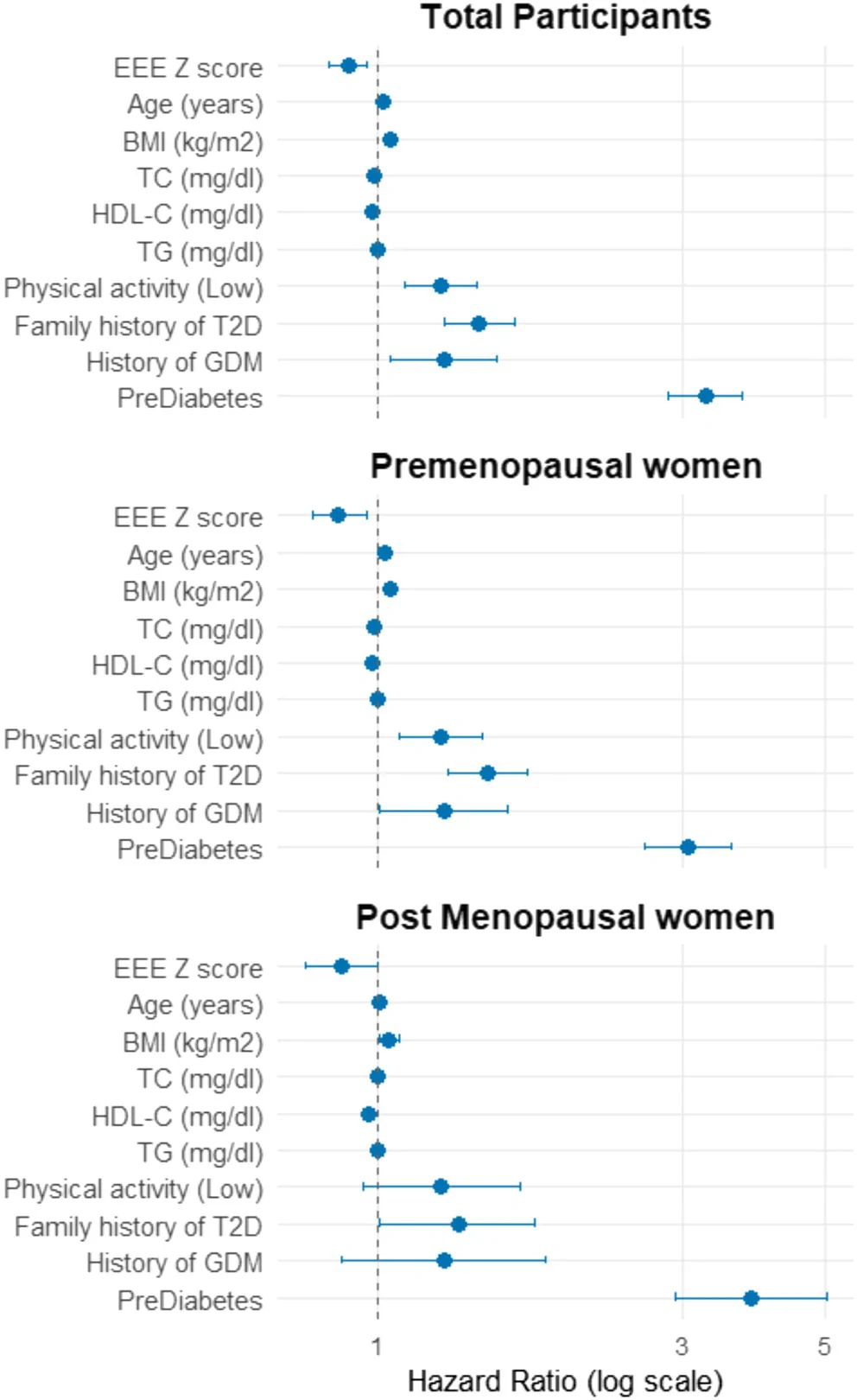
Forest Plot of the associations between EEE Z‐Score and incident T2D in total participants and across menopausal Subgroups.

Figure 3 displays the adjusted hazard ratios and their confidence intervals, providing an integrated visualisation that supports comparisons across the total, premenopausal, and postmenopausal groups.

## 4 Discussion

The present study indicates that a reduction in EEE duration is significantly associated with an increased risk of T2D in women, particularly among premenopausal women, where this correlation is especially pronounced.

It is important to note that in the subgroup of menopausal women, a reverse association between EEE duration and T2D incidence was marginally significant because the sample size in this group was small. Several studies have shown that reproductive factors can significantly impact T2D risk. These factors exert distinct effects on T2D; for instance, longer durations of breastfeeding have been associated with a decreased risk, whereas parity may potentially increase the risk [12, 23, 24]. Additionally, lifelong use of hormonal contraception may directly influence CVD risk factors, including T2D incidence, although the evidence regarding this association remains conflicting [25, 26, 27]. The relationship between the duration of reproductive periods defined by menarche and menopause and T2D remains inconclusive due to conflicting evidence [9, 28, 29]. It has been proposed that a comprehensive assessment of each woman's cumulative reproductive events is essential for gaining a thorough understanding of her risk profile for NCDs [30]. Despite this, the influence of women's cumulative reproductive histories on the incidence of T2D remains poorly understood and warrants further investigation. There is a notable gender disparity in the incidence of diabetes during both reproductive and menopausal periods. Specifically, men tend to receive a diagnosis at a younger age and with lower body fat mass compared to women. On a global scale, it is estimated that approximately 17.7 million more men than women are affected by diabetes mellitus, while the prevalence of diabetes among women significantly increases after menopause and in older age [31, 32]. Furthermore, young women with T2D are currently less likely than men to receive the treatment and CVD risk reduction recommended by guidelines [23]. One of the key components of metabolic syndrome is insulin resistance, a condition that significantly elevates the risk of developing T2D and CVD [33]. The available evidence suggests that oestrogen plays a crucial role in modulating glucose balance by enhancing and/or adjusting insulin sensitivity in various tissues and organs [34]. Oestrogen is known to influence various insulin‐sensitive tissues by activating oestrogen receptors in the brain, liver, skeletal muscle, adipose tissue, and pancreatic beta cells [35]. However, the specific roles of these tissues and the underlying mechanisms remain to be elucidated [35]. This hormonal influence underscores the complex interplay between reproductive factors and metabolic health, highlighting the need for gender‐specific approaches to understanding diabetes risk.

Menopause significantly influences the metabolic status of women, increasing the risk of developing T2D. Before menopause, oestrogen helps prevent T2D by enhancing insulin sensitivity and glucose‐stimulated insulin secretion, and by reducing beta‐cell apoptosis [36, 37]. Oestrogens may prevent the onset of insulin resistance by affecting the metabolic mechanisms related to energy equilibrium and suppressing inflammation [33, 38]. Generally, sex hormones appear to significantly impact energy metabolism, body composition, vascular function, and inflammatory responses [39]. Overall, oestrogen is protective in women's metabolic health, particularly in the response to glucose across various glucose‐sensitive tissues and organs [40]. Consequently, reductions in oestrogen can dramatically affect energy metabolism and overall metabolic homeostasis [33]. Additionally, menopause is linked to an androgenic shift that increases bioavailable testosterone due to lower oestrogen levels, promoting visceral fat accumulation and further contributing to insulin resistance [41]. Moreover, women frequently experience weight gain during menopause, influenced not just by menopause itself but also by broader lifestyle and ageing factors, thus exacerbating metabolic risks [41, 42]. Furthermore, the metabolic changes associated with menopause elevate the risk of cardiovascular diseases, which often coexist with T2D [15, 43].

The baseline differences between participants who developed T2D and those who did not are consistent with established metabolic and demographic risk factors for diabetes. These variables were included in the multivariable models to account for potential confounding. Importantly, the association between EEE and incident T2D remained after adjustment, suggesting that the observed relationship was not explained by these baseline differences.

Although our findings indicate that longer EEE is associated with a reduced risk of developing type 2 diabetes (T2D), it is important to interpret these results alongside evidence demonstrating adverse metabolic effects of oestrogen in type 1 diabetes and insulin‐deficient animal models [44, 45]. These discrepancies likely reflect fundamental differences in underlying pathophysiology. In type 1 diabetes, oestrogen acts within an autoimmune and insulin‐deficient environment, where alterations in inflammatorysignalling, counterregulatory hormone responses, and hepatic glucose dynamics may exacerbate metabolic instability [44, 45, 46]. In contrast, the predominant defect in T2D is insulin resistance, a state in which oestrogen is known to exert anti‐inflammatory, insulin‐sensitising, and adiposity‐modulating effects through Erα‐mediated pathways [47, 48]. Thus, the protective association observed in our cohort may reflect oestrogen's favourable actions in insulin‐resistant metabolic states, whereas its effects may differ substantially in the context of absolute insulin deficiency. Further studies are needed to clarify how hormonal context and diabetes phenotype modify the metabolic consequences of oestrogen exposure.

### 4.1 Strengths and Limitations

The present study is among the most comprehensive population‐based prospective studies, featuring long‐term follow‐up, a large sample size, and precise estimation of T2D. This research enables us to conduct survival analysis, account for significant potential confounding factors, and evaluate the impact of EEE duration on the risk of developing T2D.

It is essential to acknowledge the limitations of the current study, including the potential for recall bias. Data regarding the ages of menarche and menopause, as well as the initiation and conclusion of breastfeeding and the duration of hormonal contraception usage, were all self‐reported. However, within the TLGS cohort, this information was recorded every three years, demonstrating good consistency. When calculating the duration of the estradiol‐dominant (follicular) phases of menstrual cycles, the two‐week progesterone‐dominant (luteal) phase was subtracted, which may limit the estimation of the follicular phase for all participants [15, 21]. As anticipated, the absence of random error does not affect point estimation and cannot introduce bias into the results. Additionally, it is important to note that this study was conducted in an urban area, which may limit the applicability of the conclusions to rural locations. Furthermore, since the study was retrospective, the findings should be interpreted as associations rather than causal relationships.

Another limitation of our study is that we did not evaluate whether the association between oestrogen exposure and metabolic health differs in populations with autoimmune diabetes. Prior evidence suggests that oestrogen may exert adverse metabolic effects in the context of type 1 diabetes and insulin deficiency [45, 46]. Although our cohort predominantly reflects risk factors for T2D, future research should investigate whether EEE influences metabolic trajectories differently across distinct diabetic phenotypes.

Furthermore, fasting insulin measurements required to calculate HOMA‐IR were not available in our dataset; therefore, this measure was not included in the present analysis.

In conclusion, our findings suggest that prolonged exposure to EEE may be associated with a lower hazard of T2D. This insight can guide targeted interventions and clinical trials for women with shorter EEE exposure, enhancing personalised preventive care strategies by integrating EEE duration into T2D screening tools. This approach may reduce T2D risk, which is a crucial factor in the development of cardiovascular disease among these women.

Future research should focus on clarifying how EEE duration affects T2D risk to aid in the development of primary prevention strategies and to identify female‐specific factors contributing to T2D.

## Author Contributions

**Fatemeh Mahboobifard:** software. **Mahbanoo Farhadi‐Azar:** resources. **Maryam Farahmand:** conceptualization, investigation, funding acquisition, writing – original draft, methodology, validation, visualization, writing – review and editing, software, project administration, supervision, resources. **Maryam Mousavi:** formal analysis, data curation. **Fereidoun Azizi:** supervision. **Fahimeh Ramezani Tehrani:** writing – review and editing.

## Funding

This study was funded by the Research Institute for Endocrine Sciences, Shahid Beheshti University of Medical Sciences, Tehran, Iran (Grant No. 3–43010708).

## Ethics Statement

This study was conducted in compliance with the principles outlined in the Declaration of Helsinki and received ethical approval from the institutional ethics review board of the Research Institute for Endocrine Sciences, Shahid Beheshti University of Medical Sciences, Tehran, Iran, under approval number IR.SBMU.ENDOCRINE.REC. 1404.008. Before the study began, all participants gave written informed consent.

## Conflicts of Interest

The authors declare no conflicts of interest.

## Data Availability

The data that support the findings of this study are available on request from the corresponding author. The data are not publicly available due to privacy or ethical restrictions.
